# Regulatory Ouabain Action on Excitatory Transmission in Rat Hippocampus: Facilitation of Synaptic Responses and Weakening of LTP

**DOI:** 10.3390/biom15091236

**Published:** 2025-08-27

**Authors:** Yulia D. Stepanenko, Dmitry A. Sibarov, Sergei M. Antonov

**Affiliations:** Sechenov Institute of Evolutionary Physiology and Biochemistry of the Russian Academy of Sciences, Torez pr. 44, Saint-Petersburg 194223, Russia; juli@unixway.org (Y.D.S.); dsibarov@gmail.com (D.A.S.)

**Keywords:** ouabain, Na/K-ATPase, LTP, neurons, synaptic transmission, plasticity

## Abstract

Cardiotonic steroids (CTS), including the endogenous compound ouabain, modulate neuronal Na/K-ATPase (NKA) activity in a concentration-dependent manner, affecting neuronal survival and function. While high concentrations of ouabain are neurotoxic, endogenous levels of 0.1–1 nM exert neuroprotective effects and influence intracellular signaling. However, the effects of physiologically relevant ouabain concentrations on excitatory synaptic transmission remain unclear. In this study, we examined how 1 nM ouabain affects synaptic responses in rat hippocampal CA1 neurons. Using whole-cell patch-clamp recordings of evoked excitatory postsynaptic currents (EPSCs) and extracellular recordings of field excitatory postsynaptic potentials (fEPSPs), we found that ouabain enhances excitatory synaptic transmission, increasing EPSC amplitude and fEPSP slope by 35–50%. This effect was independent of NMDA receptor (NMDAR) activity. Ouabain reduced the magnitude of NMDAR-dependent long-term potentiation (LTP), but still augmented fEPSPs when applied after LTP induction. This implies separate additive mechanisms. These observations exhibit that ouabain, at concentrations corresponding to endogenous levels, facilitates basal excitatory synaptic transmission while partially suppressing LTP. We propose that ouabain exerts dual modulatory effects in hippocampal networks via distinct synaptic mechanisms.

## 1. Introduction

Cardiotonic steroids (CTS), including ouabain, are best known as highly specific inhibitors of the enzymatic activity of Na/K-ATPase (NKA), which causes cytotoxicity in all cells because of electrogenesis disruption. The NKA isoforms expressed in the CNS differ in their contribution to excitotoxicity because of the differences in rat isoform IC_50_s for ouabain of 48 µM, 58 nM, and 6.7 nM for α1, α2, and α3, respectively [1]. Despite these differences in IC_50_ values, all subunits are subjected to regulation by nanomolar ouabain concentrations [2]. For example, ouabain causes a neurodegeneration of cortical neurons in rat primary culture already at 10 nM, whereas the concentrations below 3 nM are neuroprotective [3,4]. In this respect, the α3 isoform appears to be of most interest, since the gap between neurotoxic and neuroprotective actions is narrow, suggesting a possible interplay of 0.5 to 1 nM ouabain found in the cerebrospinal fluid with both processes [5,6]. This makes the α3 isoform a preferable target for regulation. Endogenous CTSs, like ouabain and marinobufagenin, are synthesized in various tissues, including the adrenal glands, the hypothalamus, and the heart. Their production is regulated by physiological stressors, such as hypoxia, high sodium intake, and angiotensin II [7].

Many regulatory effects of ouabain are known. Low ouabain concentrations, particularly in the subnanomolar range (0.1–1 nM), appear to enhance NKA transport activity by 15–20% [2,8] and promote neuroprotective intracellular signaling pathways [4,9]. In excitotoxic conditions caused by overactivation of glutamate receptors, these levels of ouabain have been reported to reduce Ca^2+^ accumulation in neurons and brain slices [4,9,10]. Subnanomolar ouabain binding to NKA also activates a signaling complex, which includes Src kinase, epidermal growth factor receptor, phospholipase C, IP signaling, and MAPK/ERK cascade (for review, [2]). By a combination of these effects, endogenous ouabain may help to stabilize intracellular Ca^2+^ levels and maintain expression of anti-apoptotic proteins such as Bcl-2, thereby preserving mitochondrial function under excitotoxic conditions as a neuroprotector [4,9].

The subject of our interest, how subnanomolar ouabain could affect excitatory synaptic transmission in the CNS, is still poorly understood. There is evidence indicating that low ouabain concentrations modulate excitatory synaptic transmission. For example, exposure to 50 nM ouabain suppresses long-term potentiation (LTP) in the rat hippocampus, likely due to reduced NMDA receptor (NMDAR)-mediated calcium influx [11], a key mechanism for LTP induction [12]. Regardless of this valuable observation, the ouabain concentration in this study exceeds the endogenous concentration by at least 50 fold. Therefore, it remains unclear if physiologically relevant ~1 nM ouabain is enough to modulate excitatory synaptic transmission. For several reasons, 1 nM seems to represent an optimal concentration to evaluate ouabain regulatory signaling, which is as follows: α1 remains unaffected, α2 is well below its IC_50_ (58 nM), and α3 is only partially occupied (IC_50_ ≈ 6.7 nM) by 1 nM ouabain [1]. Therefore, this concentration is within a physiological, noninhibitory window—below neurotoxic levels.

Here, we addressed this important point and specifically investigated whether 1 nM ouabain modulates rat hippocampal excitatory synaptic transmission. Ouabain’s effects on excitatory postsynaptic responses, including whole-cell evoked excitatory postsynaptic currents (EPSCs) and field excitatory postsynaptic potentials (fEPSPs), as well as on LTP induction following theta-burst stimulation, were investigated.

## 2. Materials and Methods

### 2.1. Hippocampal Slice Preparation

All animal experiments described in this manuscript were approved by the Sechenov Institute of Evolutionary Physiology and Biochemistry Bioethics Committee (protocol no. 1–7/2022, 27 January 2022). At the age of 21–24 days, Wistar line rats (a total of 32 animals of both sexes, see Table 1) were decapitated, and their brains were rapidly removed and placed in ice-cold ACSF to obtain coronal slices from the dorsal hippocampus, 400 μm thick. We used a Leica VT1000S vibratome for cutting slices. Slices were cut at intermediate dorsal levels (–2.5 to –5.0 mm from bregma). The solution for cutting slices was sucrose-based and composed of 87 mM NaCl, 25 mM NaHCO_3_, 25 mM glucose, 75 mM sucrose, 2.5 mM KCl, 1.25 mM NaH_2_PO_4_, 0.5 mM CaCl_2_, 7 mM MgCl_2_, 0.5 mM Na-ascorbate, and 2 mM Na-pyruvate. The solution was bubbled with a gas mixture of 95% O_2_ and 5% CO_2_. Before the experiments, slices were incubated in an ACSF composed of 125 mM NaCl, 25 mM NaHCO_3_, 2.5 mM KCl, 2 mM CaCl_2_, 1.25 mM NaH_2_PO_4_, 1 mM MgCl_2_, 10 mM L-glucose, 0.5 mM Na-ascorbate, and 2 mM Na-pyruvate and bubbled with a gas mixture of 95% O_2_ and 5% CO_2_. Slices were incubated for 1 h at 35 °C and then kept at room temperature before the experiment. Fifty μM picrotoxin were added before the experiment to block GABA_A_ inhibitory synaptic transmission. Experiments were performed at room temperature (22–25 °C).

### 2.2. Recording of Evoked Postsynaptic Responses

**Figure 1:**
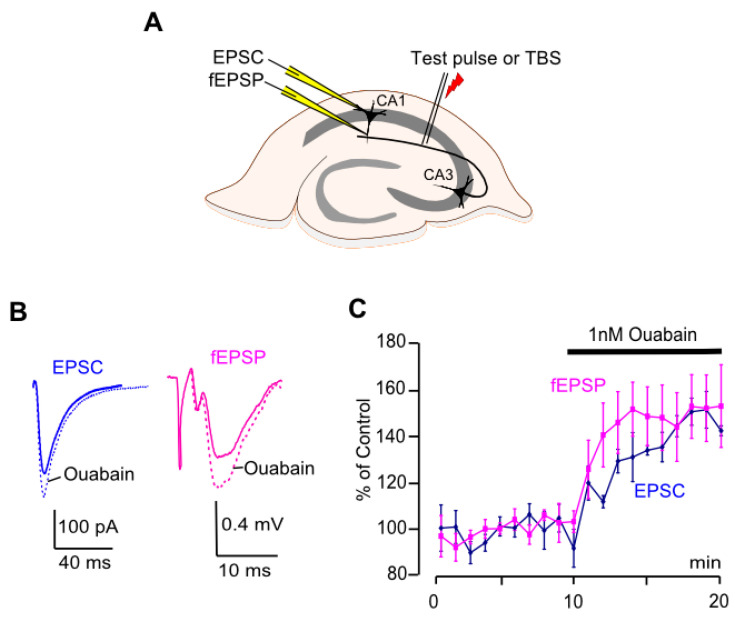
Ouabain augments EPSC amplitudes and fEPSP slopes in CA1 neurons. (**A**) Schematics of electrode positioning in the hippocampus for EPSC and fEPSP recordings. (**B**) EPSCs and fEPSPs averaged from 5 min intervals before (solid line) and after (dotted line) ouabain treatment. (**C**) Developments of the effects of ouabain (1 nM) on EPSC amplitudes and fEPSP slopes. EPSCs were recorded at −75 mV. Mean ± SEM values from field potential recordings (*n =* 7) and current recordings in pyramidal neurons (*n =* 5) are indicated. fEPSP slopes and EPSC amplitudes are normalized to control values obtained before ouabain was applied.

Slices were transferred to the recording chamber with constant oxygenated ACSF with a flow rate of 6 mL/min at room temperature. To evoke postsynaptic responses, Shaffer’s collaterals were stimulated in the area between CA1 and CA2 in stratum radiatum by a bipolar silver-wired electrode (Figure 1A). Shaffer’s collaterals were stimulated every 10 s with a 0.2 ms pulse (100–500 μA) using an A365 stimulus isolator (World Precision Instruments, Sarasota, FL, USA). fEPSPs and EPSCs were recorded using Clampex 10.3 software with a MultiClamp 700A amplifier and digital converter Digidata 1322A series (Molecular Devices, San Jose, CA, USA).

fEPSPs were recorded in CA1 stratum radiatum by a glass microelectrode filled with ACSF (~1 MΩ resistance) in current-clamp mode. EPSCs were recorded from bodies of CA1 pyramid neurons in whole-cell patch configuration at a −75 mV holding potential. The patch pipette was filled with a K^+^-based solution composed of 9 mM NaCl, 17.5 mM KCl, 121.5 mM K-gluconate, 1 mM MgSO_4_, 10 mM HEPES, 0.2 mM EGTA, 2 mM MgATP, and 0.5 mM NaGTP.

### 2.3. LTP Induction

LTP was induced by the theta-burst stimulation protocol (TBS)—a train of four 0.2 ms pulses at 100 Hz every 200 ms, repeated 10 times. TBS was applied after a stable 20 min of fEPSP or EPSC recording. The amplitude of stimulation was adjusted to evoke a response of 40–50% of maximum. For whole-cell path recordings during TBS stimulation, the amplifier was temporarily switched to current-clamp mode to allow for membrane voltage fluctuations.

### 2.4. Data Analysis

EPSC amplitudes and fEPSP slopes were analyzed in Clampfit 10.6 software. fEPSP slopes were measured as the maximal slope of the ascending phase between 20 and 70% of the fEPSP. The magnitude of any of the effects studied was estimated as the ratio of the average values after exposure to those before exposure. The data are presented as representative measurements, as well as mean values ± standard error of the mean (SEM). The sample number (*n*) refers to the number of recorded slices. Table 1 indicates the total number and gender of the animals used to obtain the datasets. Male and female animals were pooled together for analysis.

GraphPad Prism software v9.0 was used for data analysis. The normality test was performed using the Shapiro–Wilk test (*p* < 0.05) (Table 1). Groups with normally distributed data were compared using Student’s *t*-test. If at least one group did not follow a normal distribution according to the Shapiro–Wilk test, the comparison was performed using the Mann–Whitney test, which follows a common practice for such studies [13]. Data was considered significantly different with ‘*p*’ values below 0.05. Data from both male and female rats were combined for statistical analysis.

### 2.5. Drugs

Compounds were acquired from Sigma-Aldrich, St. Louis, MO, USA, particularly D-(–)-2-Amino-5-phosphonopentanoic Acid (AP5) A8054, Na-ascorbate PHR1279, Picrotoxin P1675, Na-pyruvate P2256, and Ouabain octahydrate O3125.

## 3. Results

### 3.1. Ouabain Increases EPSC Amplitude and fEPSP Slope in CA1

To evoke EPSCs and fEPSPs, Shaffer’s collaterals were stimulated in the area between CA1 and CA2 (Figure 1A). EPSCs evoked every 10 s were recorded in the pyramidal neurons at −75 mV, which prevented activation of voltage-gated channels and ensured the Mg^2+^ block of NMDARs. Ouabain at 1 nM caused a considerable 35% increase (*p* < 0.0001) of the EPSC amplitude in the pyramidal neurons of the hippocampus CA1 area (Figure 1B,C; Table 2). Consistently, a 50% increase in slope (*p* < 0.01), which, as it is generally interpreted, results from the increase of amplitudes of excitatory synaptic responses in a population of neurons recorded by a field electrode, was observed for fEPSPs in the CA1 area (Figure 1B,C; Table 2).

Coincidences of observations obtained on the EPSC and fEPSP validate the data and support the utility of both experimental approaches for these sorts of measurements. Whereas patch-clamp recordings allow measurements on a single neuron, field potentials reflect the ouabain effect on a population of neurons. Overall, neither changes in membrane potential nor voltage-gated channels of postsynaptic neurons are involved in this effect. Unfortunately, we did find references that ouabain may directly influence the amplitude of AMPAR responses on a time scale of 1–2 min, the range that we observed in the experiments. At long-lasting measurements of about an hour, however, low ouabain doses did not affect AMPAR expression [14], and at high doses, the expression was even reduced [15]. We, therefore, cannot exclude that the increase of excitatory synaptic responses caused by ouabain is determined by presynaptic action, which was previously demonstrated by a miniature postsynaptic current in neuronal cultures and brain slices [4,10].

### 3.2. Ouabain-Induced Rise of fEPSP Slope Is NMDAR-Independent

**Figure 2:**
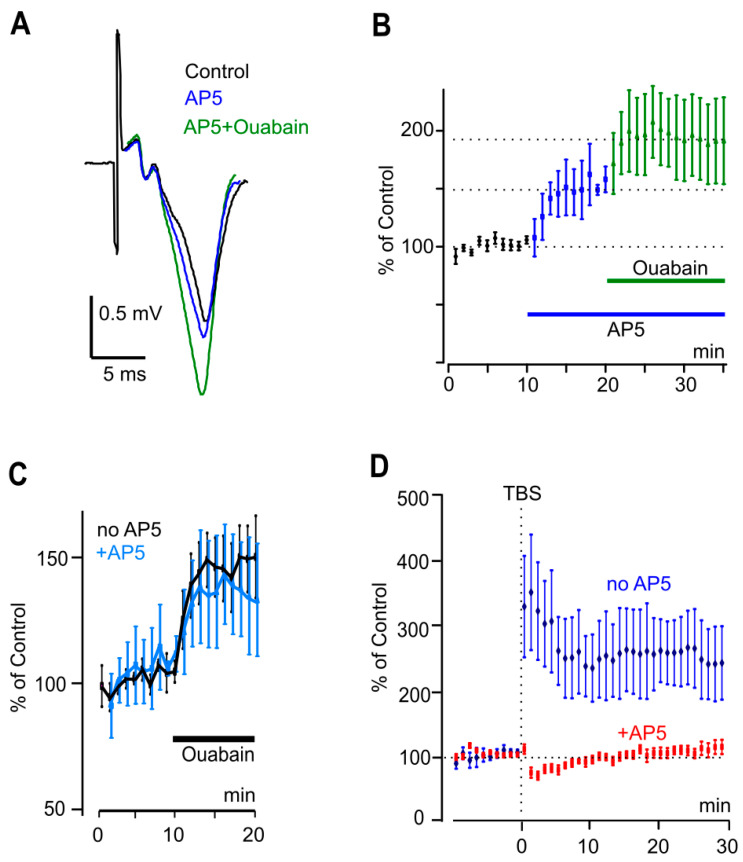
A role of NMDARs in ouabain effects on LTP and fEPSPs. (**A**) fEPSPs averaged for 5 min in control (black), upon 50 µM AP5 application (blue), and upon AP5 + 1 nM ouabain application (green) recorded from the same slice. (**B**) Development of the effects of AP5 (50 µM) and ouabain (1 nM) on fEPSP slopes. Mean ± SEM values of slopes from experiments (*n =* 6). Slopes are normalized to control values obtained before AP5 was applied. The intervals of application of reagents are shown as bars below the traces. (**C**) Development of the effects of ouabain (1 nM) on EPSP slopes in the absence (black) and in the presence (blue) of 50 µM AP5. Mean ± SEM of fEPSP slope from experiments in the absence (*n =* 7) and the presence (*n =* 6) of AP5 measured before and after ouabain. Slopes are normalized to control values obtained before ouabain was applied. (**D**) Development of LTP induced by TBS in the absence (blue, *n =* 7) or in the presence (red, *n =* 8) of 50 µM AP5. Mean ± SEM of slopes are normalized to values before TBS.

As far as ouabain reduces the calcium responses induced by glutamate receptor activation [4,10], one may assume that the reduction of presynaptic and postsynaptic intracellular calcium may contribute to the effects on synaptic transmission. Therefore, the possible contribution of NMDARs to ouabain’s effects was investigated. To test whether the increase in fEPSP slope involves an interaction between NKA and NMDARs, 50 µM AP5 (an NMDAR antagonist) was added 10 min prior to ouabain. It should be noticed that AP5 increased the fEPSP slope by itself (Figure 2A,B). This observation coincided with previously described research that NMDAR inhibition attenuates spontaneous miniature EPSPs but potentiates evoked fEPSPs [16]. Nevertheless, under the NMDAR block, ouabain caused an additional 34% increase in the fEPSP slope (*p* < 0.002) (Figure 2B,C; Table 2). Therefore, both in the absence and in the presence of AP5, the degree of synaptic transmission facilitation by ouabain revealed by an analysis of fEPSP slopes was similar (Figure 2C). Thus, the ouabain-induced facilitation of excitatory synaptic transmission in the hippocampus did not depend on NMDARs. To further characterize excitatory synaptic transmission, we performed experiments in which the development of LTP was analyzed. TBS protocol induced typical LTP of excitatory synaptic transmission in pyramid neurons with a 150% increase of fEPSP slope (Figure 2D). AP5 abolished LTP formation, suggesting that this LTP is NMDAR-dependent (Figure 2D).

### 3.3. Ouabain Reduces LTP

**Figure 3:**
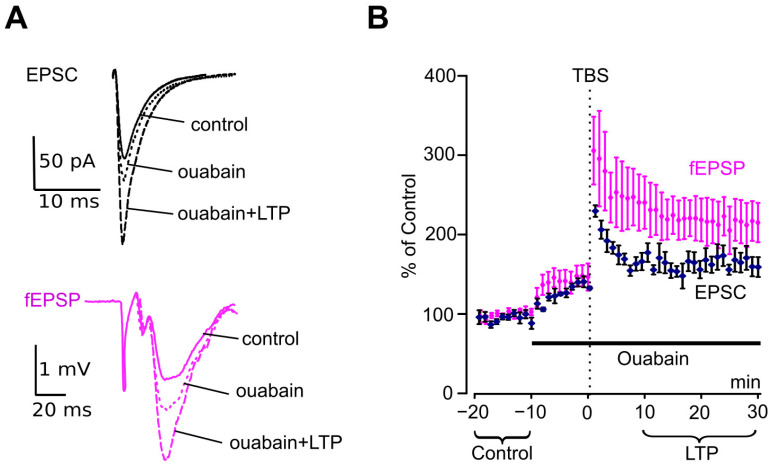
LTP induction and development in the presence of ouabain. (**A**) EPSCs recorded at −75 mV and fEPSPs averaged for intervals before (solid line) and after application of 1 nM ouabain but before TBS (dotted line) and after LTP induction in the presence of ouabain (dashed line). (**B**) Development of the effects of ouabain (1 nM) and TBS on EPSC amplitudes (black) and fEPSP slopes (purple). Mean ± SEM of experiments in slices (*n =* 5) and in pyramidal neurons (*n =* 7) are indicated. Slopes and amplitudes are normalized to control values obtained before ouabain was applied.

Given ouabain’s effects on NMDAR-mediated intracellular Ca^2+^ signaling [4,9,10,11], it remains unclear whether ouabain may affect LTP, which depends on NMDAR activation. Experiments were performed both by patch-clamp recording of EPSCs and by extracellular recording of fEPSPs. Under control conditions, when EPSCs recorded at −75 mV were evoked with a rate of stimulation of 1 per 10 s, their amplitudes were rather stable (Figure 3A,B). Ouabain at 1 nM caused a typical increase in the EPSC amplitude. The TBS protocol applied in the presence of ouabain caused an additional considerable increase in EPSC amplitudes, suggesting LTP formation (Figure 3A,B). The amplitude of EPSCs measured at the LTP stable level was twice as large as the amplitude under the control condition (Figure 3A). For fEPSP recordings, the dynamics of slope change over time in response to ouabain and TBS were quite similar to those of the EPSC amplitude (Figure 3A,B). This strongly suggests that the response to ouabain and LTP dynamics coincide for single neurons and the large neuronal population, which exhibits synaptic strengthening across the network. Our experiments demonstrated that extracellular fEPSP recordings are representative and appropriate for our further investigation of ouabain’s effects on LTP.

**Figure 4:**
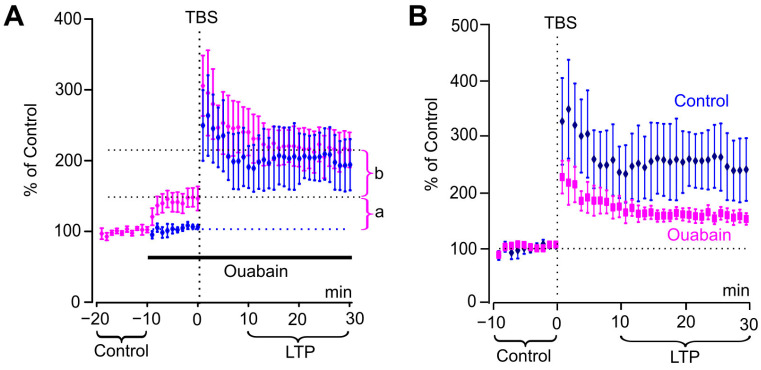
Ouabain weakens LTP. (**A**) Development of the effects of ouabain (1 nM) and LTP on fEPSP slopes, which are normalized to values before application of ouabain. TBS-induced LTP is shown in control (blue) and upon ouabain addition 10 min prior to TBS (purple). Mean ± SEM of slopes from experiments without (*n =* 7, blue) and with ouabain (*n =* 7, purple) are indicated. a—ouabain-induced increase of fEPSP slope. b—additional LTP-induced increase of fEPSP slope in the presence of ouabain. (**B**) The same data as on (**A**), but fEPSP slopes are normalized to a 10 min interval before TBS to evaluate pure LTP-induced facilitation of fEPSP slope.

In subsequent experiments, we addressed the question of how ouabain affects the TBS-induced increase of the fEPSP slope. Without ouabain treatment, LTP increased the fEPSP slope to 250 ± 57% (*n =* 7) of that before TBS. In consistency with previous experiments, when 1 nM ouabain was applied prior to the TBS protocol, it caused an increase in the fEPSP slope (Figure 4A, fraction a). TBS further increased the fEPSP slope (Figure 4A, fraction b), reaching 230 ± 30% (*n =* 7) of control. The final fEPSP facilitation 10–30 min after TBS was not different between the ouabain-treated and -untreated slices (Figure 4A).

Therefore, in slices treated with ouabain, the total increase in fEPSP slope after LTP induction reflected the combined effects consisting of two components: (a) an NMDA receptor-independent potentiation caused by ouabain and (b) NMDA receptor-dependent LTP (Figure 4A, components a and b, respectively). To emphasize the pure ouabain effect on LTP, we reanalyzed the data (from Figure 4A) by taking the fEPSP slope in the presence of ouabain (prior to TBS) as 100% (Figure 4B). This analysis demonstrated that, in ouabain-treated slices 10–30 min after TBS, solely LTP increases fEPSP slope to 150 ± 12% of control, which is significantly lower than the 250% value observed in ouabain-untreated slices (*n* = 7, Mann–Whitney test, *p* < 0.02).

Based on these results, we hypothesized that ouabain produces two distinct effects, and that the effects of ouabain on LTP and fEPSPs are additive. Whether ouabain is able to alter the fEPSP slope after the onset of LTP was examined in further experiments.

### 3.4. Ouabain Increases fEPSP Slope After LTP Induction

**Figure 5:**
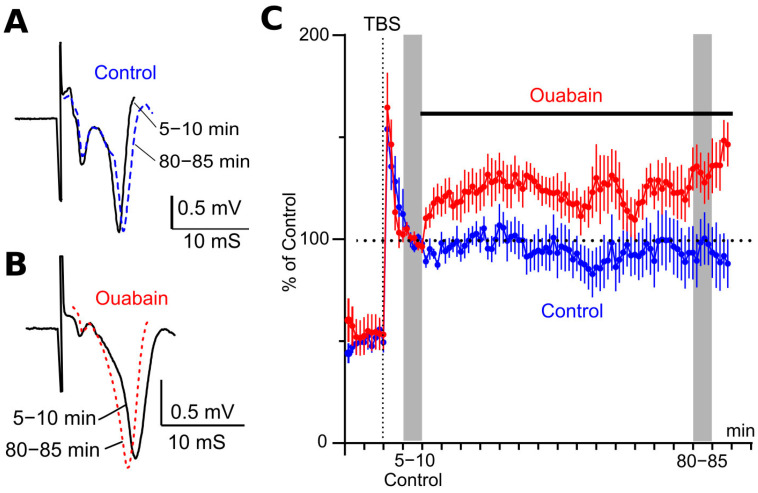
Ouabain facilitates fEPSP after LTP induction. (**A**) fEPSPs recorded in ouabain-untreated slices (*n =* 5) and averaged for intervals 5–10 min after TBS (solid line) and 80–85 min after TBS (dotted line). (**B**) Same as (**A**), but for slices where 1 nM ouabain was present at 80–85 min interval (*n =* 7). (**C**) Developments of the effect of ouabain (1 nM) on fEPSP slopes after induction of LTP. The change of slope is shown in the control (blue) and with the addition of ouabain 5 min after TBS (red). Mean ± SEM of slopes from experiments in ouabain-treated (*n* = 7, red) and untreated (*n* = 5, blue) slices. Slopes are normalized to 5–10 min interval after LTP induction, but before application of ouabain. The application of ouabain is shown as a horizontal bar above the traces.

Typically, after induction of LTP by TBS, the slope of evoked responses gradually decreases over 30–40 min and then stabilizes at a constant level [11,17,18,19]. In our experiments, during an interval of 5–10 min after TBS, fEPSPs demonstrated a similar shape. Their average value of slopes was picked as a reference. fEPSPs remained stable through the duration of the experiments so that recordings in the interval 80–85 min were similar to the fEPSPs obtained in the reference interval (Figure 5A). When 1 nM ouabain was applied 10 min after TBS, the fEPSP slope increased, as it became obvious from the difference of fEPSP shapes recorded before and at the interval of 80–85 min in the presence of ouabain (Figure 5B). Overall, ouabain increased the fEPSP slope by 33% compared to the untreated slices (*p* < 0.04) at the 80–85 min time interval (Figure 5C; Table 2).

Thus, from these experiments, we can conclude that ouabain augments the slope both before and after LTP induction to approximately the same extent. The observed ouabain-induced fEPSP potentiation seems to be NMDA-independent and is probably not associated with the LTP induction mechanism.

## 4. Discussion

### 4.1. Effects of Ouabain on Evoked Synaptic Responses

Here, we demonstrate that, in CA3–CA1 synapses, 1 nM of ouabain increased synaptic responses. As ouabain may influence NKA ion transport even at low nanomolar concentrations [2], some changes of postsynaptic membrane potential in the presence of ouabain are expected. Our observation that 1 nM ouabain increases EPSCs recorded in CA1 neurons voltage-clamped at −75 mV avoids the conditions at which any changes of membrane voltage may occur. Therefore, the ouabain effect can be attributed neither to a change in postsynaptic membrane voltage nor to modulation of Ca^2+^ entry via voltage-gated channels or NMDA receptors, which undergo Mg^2+^ block at −75 mV [20,21]. In addition, at −75 mV, in CA1 neurons, voltage-dependent Ca^2+^ entry to postsynaptic regions is prohibited, which excludes the calcium-triggered upregulation of postsynaptic AMPARs [22]. Unfortunately, our experiments do not allow us to analyze the mechanisms of action of ouabain. AMPA receptors are subjected to both rapid modulation by polyamines [23] and slower processes of trafficking, calcium-dependent phosphorylation, and transient incorporation of GluA2-lacking AMPA receptors [24,25]. Considering the rate of development of the ouabain effects on synaptic responses in our experiments (1–2 min), it seems unlikely that AMPAR conductance or open probability could be affected by ouabain via CaMKII, PKA, or PKC phosphorylation. It becomes clear that an activation of these kinase pathways by NKA signaling typically requires several hours [9]. In addition, low doses of ouabain do not alter AMPAR expression [14]. Since the regulation of AMPA receptors by ouabain is currently unknown, we cannot exclude possible presynaptic action from consideration.

The increase in synaptic strength by both ouabain and LTP poses the question of whether the two phenomena are independent. The LTP formation in our experiments was prevented by NMDAR block, while ouabain-induced facilitation of synaptic responses was not. Whereas LTP induction required postsynaptic depolarization [26], ouabain effects did not. Moreover, ouabain increased postsynaptic responses after LTP onset, producing additive amplification of postsynaptic responses.

The precise signaling pathways activated by ouabain in the presynaptic terminal remain unclear. However, several potential mechanisms may explain the ouabain-induced enhancement of neurotransmitter release from the presynapse. First, in nerve terminals, 3 nM ouabain augments the releasable pool of Ca^2+^ stored in the neuronal endoplasmic reticulum and increases stimulus-evoked calcium transients [27], which are the main drivers of synaptic vesicle release. This facilitation of intracellular Ca^2+^ responses did not require NMDAR activity [27], which matches our electrophysiological data on the NMDAR-independent facilitation of postsynaptic responses by ouabain. Hence, an ouabain-induced increase in presynaptic Ca^2+^ transients may potentiate stimulus-elicited glutamate release, leading to amplification of postsynaptic AMPAR currents.

The second possible mechanism is the ouabain-mediated suppression of spontaneous miniature postsynaptic currents [4,10], which originate from asynchronous spontaneous synaptic vesicle release. Suppression of asynchronous release by ouabain [4,10] may engage more vesicles to be available for action potential-evoked transmitter release because these mechanisms share the same readily releasable pool of synaptic vesicles [28,29]. One nM ouabain decreases cytosolic Ca^2+^ [4,10], suggesting that asynchronous release is reduced and local Ca^2+^ dynamics may favor a sharper, more synchronized response to the action potential.

### 4.2. LTP Inhibition by Ouabain

We observed an attenuation of LTP-induced facilitation of postsynaptic responses in the presence of 1 nM ouabain that aligns with prior findings by Akkuratov et al. [11] and supports the hypothesis that ouabain modulates synaptic plasticity exclusively through the interaction with the only molecular target found, NKA. Multiple signaling pathways might be involved in ouabain-induced suppression of LTP.

During LTP induction, ouabain may influence local signaling complexes involving NKA, the sodium–calcium exchanger (NCX), and NMDARs. The binding of ouabain at 1 nM to NKA could stimulate NCX-driven Ca^2+^ extrusion, reducing the postsynaptic Ca^2+^ accumulation required for LTP formation. The suppression of glutamate-induced intracellular Ca^2+^ accumulation by ouabain [4,11,30,31] could be mediated through direct protein–protein interactions between NKA and NMDARs [4,11] or through functional coupling within lipid microdomains [4,30,31].

LTP development is also accompanied by a transient decrease in NKA enzymatic activity within approximately 15 min after induction [32]. This transient downregulation likely contributes to local Na^+^ accumulation, which enhances the voltage-dependent unblock of NMDARs and Ca^2+^ influx, both of which are critical for LTP consolidation [33,34]. Subnanomolar concentrations of ouabain (0.1–10 nM) may counteract this transient reduction of NKA activity due to potentiation of α1 NKA isoform enzymatic function [8,34,35,36], resulting in premature restoration of membrane resting potential, thereby limiting NMDAR activation and subsequent voltage-dependent Ca^2+^ influx.

Thus, ouabain in concentrations corresponding to the natural endogenous level affects the processes of synaptic transmission and synaptic plasticity. The hypothalamus has been specifically identified as a site of cardiotonic steroid production within the CNS [37]. Hypothalamic cells can secrete ouabain-like factors, and this secretion is stimulated by aldosterone via mineralocorticoid receptors [38]. This suggests the involvement of endogenous CTS production in stress responses. Our results highlight the role of endogenous cardiotonic steroids in neuronal excitability and synaptic strength within memory-related circuits in the hippocampus, which presumes their role in memory formation.

When considering the physiological action of cardiotonic steroids, gender differences in glutamatergic signaling [39,40] should be taken into account. In particular, pregnancy can double the concentration of circulating endogenous ouabain [41]. Hypertension also reveals the gender difference of NKA activity [42]. However, this difference manifests stronger in adults and under pathological conditions. In pre-pubertal and non-pregnant animals, the circulating levels of endogenous ouabain in both genders are similar [41,43]. To minimize the influence of animal sex, we used prepubertal rats and included males and females in each data set. Nevertheless, future studies are required to address the gender differences of cardiotonic steroid action in the CNS.

## 5. Conclusions

Our findings demonstrate that ouabain, at concentrations corresponding to endogenous levels in the CNS, modulates synaptic function in the hippocampal CA3–CA1 synapses. Specifically, 1 nM ouabain enhances evoked synaptic responses, which probably involves an increase in glutamate release. The observed effects likely reflect a complex action of ouabain at both pre- and postsynaptic sites. Nevertheless, ouabain-induced potentiation of synaptic transmission occurs independently of postsynaptic depolarization and NMDA receptor activation, distinguishing it from classical LTP mechanisms. In addition, ouabain impairs LTP induction, likely by modulating NKA signaling and interfering with the critical calcium dynamics required for synaptic plasticity. These findings raise the possibility that ouabain released during stress could help stabilize existing synaptic connections while at the same time restricting the formation of new ones. Future studies are required to investigate if endogenous ouabain might influence memory encoding and consolidation under physiologically relevant conditions.

## Figures and Tables

**Table 1:** Animal gender and dataset test for normality.

	Section in Results	Slices (*n*)	Animals	Sex	Normality
fEPSP	3.1	7	5	3 ♂ + 2 ♀	yes
EPSC	3.1	5	4	1 ♂ + 3 ♀	no
fEPSP, AP5	3.2	6	4	3 ♂ + 1 ♀	yes
fEPSP, Control	3.3	7	3	1 ♂ + 2 ♀	yes
fEPSP, with AP5	3.3	8	4	2 ♂ + 2 ♀	no
fEPSC, Ouabain pre-treat	3.3	5	3	3 ♂	yes
fEPSP, Ouabain pre-treat	3.3	7	3	2 ♂ + 1 ♀	yes
fEPSP, Control	3.4	5	3	2 ♂ + 1 ♀	yes
fEPSP, Ouabain post-treat	3.4	7	3	1 ♂ + 2 ♀	no
Total		57	32	20 **♂** + 12 **♀**	

**Table 2:** Ouabain effects on EPSC amplitude and fEPSC slope.

	Intact (fEPSP)	Intact (EPSC)	+AP5 (fEPSP)	After LTP Onset (fEPSP)
Control	99 ± 0.2 (*n =* 7)	98 ± 0.4 (*n =* 5)	102 ± 2 (*n =* 6)	95 ± 11 (*n =* 5)
Ouabain	150 ± 13 (*n =* 7) ##, *p* < 0.01	135 ± 0. 9 (*n =* 5) ****, *p* < 0.0001	134 ± 6 (*n =* 6) **, *p* < 0.002	133 ± 11 (*n =* 7) *, *p* < 0.04

Data are presented as % of control values before application of ouabain. *, **, ****—data are significantly different from control according to the Mann–Whitney test. ##—data are significantly different from control according to Student’s *t*-test.

## Data Availability

The original contributions presented in the study are included in the article; further inquiries can be directed to the corresponding author.

## Abbreviations

ACSF	Artificial cerebrospinal fluid
AMPAR	α-amino-3-hydroxy-5-methyl-4-isoxazolepropionic acid receptor
AP5	(2R)-amino-5-phosphonovaleric acid
CaMKII	Calcium/calmodulin-dependent protein kinase II
CTS	Cardiotonic steroids
EPSC	Excitatory postsynaptic current
fEPSP	Field excitatory postsynaptic potential
LTP	Long-term potentiation
NMDAR	*N*-methyl-D-aspartate receptor
NKA	Na^+^/K^+^-ATPase
PKA	Proteinkinase A
PKC	Proteinkinase C
SEM	Standard error of mean
TBS	Theta-burst stimulation
